# Notes and News

**Published:** 1890-09-20

**Authors:** 


### NOTES AND NEWS.

Collected and Communicated.

Ilfracombe collected £198 11s. 6d., as against £194 12s. last year, and it is satisfactory to note that the expenses have decreased by nearly one-half. The Jessell Cottage Hospital has thus been benefited by a cheque for £191 12s. 10d., no mean tribute to the energy of the committee.

The idea that monuments to the departed must take the form of brasses, mural inscriptions, and stained-glass windows has held its ground too long. Mr. Quilter's conception of how best to perpetuate the memory of "our beloved dead" is surely a truer one. Our notions of death and life are not so far dissociated that a home, where those who have been sick may recruit their strength to lead new lives of health and usefulness, may not be a fitting memorial to those others who have bequeathed us a high ideal of what our lives should be.

The original Hospital Saturday collection at Woolwich, made on July 5th, was so interfered with by rain that a supplementary collection has been made, resulting in a further sum of £112 16s. 8d., which, added to the previous total of £151, gives an excess of some £40 over the sums collected in the district on any previous occasion. Of the total received on this last occasion, £60 18s. 2d. was in bronze—an undeniable testimony to the value of the fund as a means of collecting the most modest subscriptions, which modesty might otherwise blush to give.

The Victoria Jubilee Hospital at Swaffham in Norfolk is to be congratulated upon its progress. Its second annual report, which we have just received, shows a balance in hand of £42 5s. 3d., after paying all expenses for the past year. During the year 50 patients have been admitted, of whom 38 were discharged cured, 7 much benefited, 1 incurable; 3 are still under treatment, and 1 died. Subscriptions produced over £118, and an Endowment Fund has now been started with a donation of twenty guineas, which the committee express a hope may soon be enlarged.

The report of the Ramsgate and St. Lawrence Dispensary is a good one. Of a total number of 2,015 patients treated during the year, 1,665 were cured, 19 relieved, 59 died, and 272 were still under treatment at the date of the report. The balance-sheet showed that the year opened with a balance in hand of £91 10s., and closed with a balance of £111 6s., in spite of an increase of patients and a severe tax on the resources of the institution. This Dispensary appears to be doing valuable work in an economical and careful manner.

The great need of isolation wards which has again been felt in the Sunderland and Bishopwearmouth Infirmary, owing to several outbreaks of erysipelas and several cases of zymotic disease, is at last to be met, Miss Forster, a principal owner of South Hetton Colliery, having generously offered to defray the cost of building such wards. Suggestions by the committee include the reconsideration of the provision of a mortuary and post-mortem room, and, further, the ultimate establishment of a convalescent home for patients passing through the infirmary.

The Devon and Cornwall Ear and Throat Hospital at Plymouth is steadily making its way and unostentatiously doing good work. The fourth annual report shows that during this last year 390 cases had been under care, of which 178 were cured, 135 much relieved, 31 were incurable, and one died. In the remaining 45 cases there were only one or two attendances which did not allow of their being successfully treated. Hitherto, the institution has been severely handicapped in dealing with grave cases where operations are required, owing to the fact that it has possessed no accommodation for in-patients, but we are glad to learn that this disadvantage is likely to be removed before very long. The financial statement shows that the balance to the credit of the hospital has been again raised, and now amounts to nearly £60.

Mr. Cuthbert Quilter's generous gift of £2,000 to build the women's wing of the Felixstowe Convalescent Home met with fullest recognition on the occasion of the opening of the new buildings last week. We can imagine few more difficult positions for a man to occupy than that in which Mr. Quilter stood that day. His desire that the donor should remain anonymous proved vain, a fact of which everyone must be glad, and the consequence was that he had not only to listen, but to reply to more pretty things in an afternoon than fall to most men's lot in a lifetime. But Mr. Quilter proved equal to the situation, and his speech, which was simple and manly, was inspired by the same true feeling that prompted the gift. Everyone will echo his prayer that the institution "may be a blessing for years to come to the sick and to those requiring rest from all parts of Suffolk."

The twentieth report of the Alfred Hospital, Melbourne, shows that during the year ending June 30th, 1890, a total of 4,351 cases received treatment, of which 1,612 were in-patients, 2,267 out-patients, and 472 casualties. Of the in-patients 1,276 were discharged cured or relieved, and 216 died, leaving 120 in residence at the date of the report. With regard to the financial position of the institution, the year which began with an overdraft of £38, ended with a deficit of nearly £450, due not to an increase of expenditure, but a serious falling off in the receipts. The maintenance account received some £1,200 less than last year, and one-half of this represents a decrease of private subscriptions. The Increased Hospital Accommodation Committee have allotted the hospital £7,000 for extensions, and this is to be expended in the erection of a new pavilion, new out-patients' department, additions to the laundry and store-room, and the conversion of the Administration Block to the purposes for which it was originally designed.

